# Optimizing Cardiovascular Assessment: Reducing Unnecessary Cardiac Troponin I Testing

**DOI:** 10.5334/gh.1421

**Published:** 2025-03-27

**Authors:** Ariana Fernandes, Aline C. T. Wisnivesky, Raíssa Rezende, Francisco A. M. Cardozo, Danielle M. Gualandro, Daniela Calderaro, Luciana Fornari, Leila Antonangelo, Nairo Sumita, Celia Strunz, Luciana D. Bichuette, Marcos P. Lottenberg, Bruno Caramelli

**Affiliations:** 1 Unidade de Medicina Interdisciplinar em Cardiologia, Instituto do Coração, Hospital das Clínicas HCFMUSP, Faculdade de Medicina, Universidade de São Paulo, São Paulo, Brasil; 2 Department of Cardiology and Cardiovascular Research Institute Basel (CRIB), University Hospital Basel, University of Basel, Spitalstrasse 2, CH-4056, Basel, Switzerland; 3 Instituto do Coração, Hospital das Clínicas, Faculdade de Medicina da USP, Brasil; 4 Laboratório Central, Hospital das Clínicas HCFMUSP, Faculdade de Medicina, Universidade de São Paulo, SP, Brazil; 5 Departamento de Patologia Clínica, LIM/03 –Laboratório de Medicina Laboratorial, Hospital das Clínicas, Faculdade de Medicina da Universidade de São Paulo, São Paulo, SP, Brasil

**Keywords:** troponin, Biomarker, Acute coronary syndrome, cost

In the last few decades, cardiologists have observed an important decrease in the fatality rate for myocardial infarction (MI) patients admitted to the hospital. Faster and more accurate diagnostic methods, precise risk stratification, and better treatment are related to this auspicious scenario (1). Cardiac troponin I (cTnI) has emerged as a critical biomarker for assessing myocardial injury, confirming diagnosis, identifying high-risk individuals, and guiding treatment.

On the other hand, despite its widespread use, the clinical application of cTnI test has also introduced challenges related to resource utilization, and cost-effectiveness. Indeed, the American Heart Association (AHA) guidelines have identified cTnI testing as an area prone to low-value care, where unnecessary tests contribute significantly to healthcare expenditure without necessarily improving patient outcomes (2).

The cost-effectiveness of managing acute cardiovascular syndromes has become increasingly important, given that cardiovascular disease (CVD) remains the leading cause of mortality worldwide. Its global burden has escalated, largely due to shifts in population demographics, including aging and overall population growth. In Brazil, the prevalence of CVD increased substantially between 1990 and 2019, affecting 6.1% of the population and representing the primary cause of death in the country. This challenge is even more pronounced in low-income regions, where resource availability and adherence to clinical guidelines vary significantly, leading to disparities in access to appropriate treatment for certain population groups (3,4).

This study focuses on comparing the practices of cTnI test at a General University Hospital (GUH) and a specialized Cardiology University Hospital (CUH). Our aim is to determine whether adhering to expert-recommended guidelines for cTnI test can reduce unnecessary exams.

We conducted a cross-sectional retrospective study analyzing all cTnI tests performed at GUH and CUH from November 2022 to March 2023. This study used fully anonymized and de-identified retrospective data, ensuring that no individual could be identified at any stage. In Brazil, according to Resolution CNS 510/2016 Institutional Review Board (IRB) approval was not required for this research. Both GUH and CUH are institutions from the same University and located nearby. GUH is a general medical facility, while CUH focuses primarily on cardiac patients. Patients who were receiving postoperative care following invasive cardiac procedures and those participating in clinical research protocols were excluded. The population was further categorized by sex, age (<60 or ≥60 years), and whether serial cTnI testing (defined as two or more sequential tests within 24 hours) was performed. Serial testing was considered appropriate when it adhered to the 4th Universal Definition of MI, which recommends monitoring for a rise or fall in troponin levels indicative of myocardial injury (5). Data were retrieved from the hospitals’ electronic health records. cTnI normal reference levels were in accordance to the manufacturer: <16 ng/L for women and <34 ng/L for men. We used the chi-square test to compare categorical variables and the Mann-Whitney U test for continuous data. A p-value of <0.05 was considered statistically significant.

A total of 5,360 cTnI tests were performed at GUH, and 8,487 tests at CUH. The demographic characteristics of the tested population were similar across both hospitals. At GUH, 46.2% of the tests were performed on women, and 60.9% were conducted on individuals aged ≥60 years. At CUH, 42.0% of the tests were performed on women, and 53.8% were conducted on individuals aged ≥60 years. A significantly higher proportion of normal cTnI test results was observed at GUH compared to CUH (70% vs. 45%, p < 0.0001). This suggests that a substantial number of tests at GUH may have been conducted on patients with a low likelihood of myocardial injury Figure 1.

**Figure 1:**
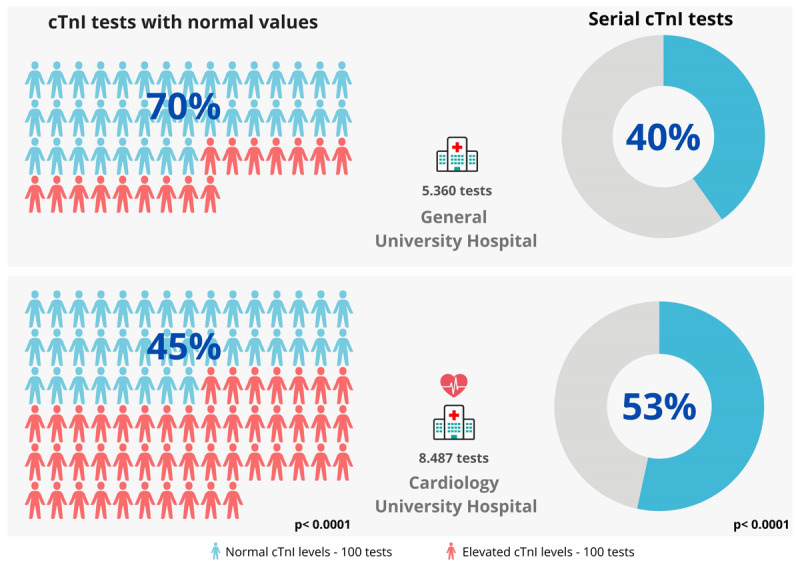
Percentage of cTnI tests with normal value and with serial cTnI tests. Legend: Comparison of cardiac troponin I (cTnI) test results between a general university hospital (GUH) and a specialized cardiology university hospital (CUH). In the GUH, 70% of tests indicated normal cTnI levels, with 40% of the tests being serial measurements. In the CUH, only 45% of tests showed normal levels, while 53% were serial measurements. The data suggest a statistically significant difference (p < 0.0001) between the two settings, indicating a higher prevalence of elevated cTnI levels and more frequent use of serial testing in the cardiology-specialized hospital.

Serial cTnI tests were less frequent at GUH than at CUH (40.3% vs. 53.4%, p < 0.0001). The lower frequency of serial testing at GUH suggests that expert-recommended guidelines, were followed less consistently in the general hospital setting Figure 1.

Median cTn I level at GUH were significantly lower than those observed at CUH (8 ng/L vs. 37 ng/L, p < 0.0001). This further supports the hypothesis that a larger proportion of tests at GUH were performed in patients with a low probability of MI, potentially resulting in inefficient test use (1,3). The higher frequency of normal cTnI test results at GUH, coupled with the lower rate of serial testing, additionally suggests that unnecessary tests may be more prevalent in general hospital environments.

Reducing low-value care has been a major focus of healthcare reform initiatives, particularly as healthcare systems worldwide face increasing pressure to improve cost-effectiveness without compromising quality. There is a notable paucity of studies evaluating the potential economic impact of reducing low-value care worldwide. Nevertheless, in the United States, the estimated annual costs associated with such care exceeded 17 billion USD in 2019 (6).

Policymakers should consider implementing hospital-specific interventions that promote adherence to best practices for cTnI testing. Education and training programs, along with decision-support tools integrated into electronic health record systems, could help guide clinicians in appropriately ordering cTnI test.

Demographic characteristics of patients included were similar in both Hospitals but the reasons cTnI for testing could be different. As an example, for patients presenting with MI symptoms at the CUH emergency room or a suspected cardiovascular complication after non-cardiac surgery at GUH. Despite this different reason for testing, the reference levels and the suspected diagnosis are the same: acute coronary syndrome. We assume that the different reason for testing would not significantly influence the interpretation of results.

## Conclusion

This study highlights the economic inefficiencies associated with unnecessary cTnI testing in a general hospital setting compared to a specialized cardiology hospital. Reducing low-value care by adhering to expert-recommended test could lead to significant cost savings and more targeted use of resources.
